# Predicting food craving in everyday life through smartphone-derived sensor and usage data

**DOI:** 10.3389/fdgth.2023.1163386

**Published:** 2023-06-26

**Authors:** Thomas Schneidergruber, Jens Blechert, Samuel Arzt, Björn Pannicke, Julia Reichenberger, Ann-Kathrin Arend, Simon Ginzinger

**Affiliations:** ^1^ Department Creative Technologies, University of Applied Sciences Salzburg, Salzburg, Austria; ^2^ Department of Psychology, Paris-Lodron-University of Salzburg, Salzburg, Austria; ^3^ Centre for Cognitive Neuroscience, Paris-Lodron-University of Salzburg, Salzburg, Austria

**Keywords:** food craving, time-lagged, prediction, ecological momentary assessment, passive sensing, personalized modeling

## Abstract

**Background:**

Food craving relates to unhealthy eating behaviors such as overeating or binge eating and is thus a promising target for digital interventions. Yet, craving varies strongly across the day and is more likely in some contexts (external, internal) than in others. Prediction of food cravings ahead of time would enable preventive interventions.

**Objective:**

The objective of this study was to investigate whether upcoming food cravings could be detected and predicted from passive smartphone sensor data (excluding geolocation information) without the need for repeated questionnaires.

**Methods:**

Momentary food craving ratings, given six times a day for 14 days by 56 participants, served as the dependent variable. Predictor variables were environmental noise, light, device movement, screen activity, notifications, and time of the day recorded from 150 to 30 min prior to these ratings.

**Results:**

Individual high vs. low craving ratings could be predicted on the test set with a mean area under the curve (AUC) of 0.78. This outperformed a baseline model trained on past craving values in 85% of participants by 14%. Yet, this AUC value is likely the upper bound and needs to be independently validated with longer data sets that allow a split into training, validation, and test sets.

**Conclusions:**

Craving states can be forecast from external and internal circumstances as these can be measured through smartphone sensors or usage patterns in most participants. This would allow for just-in-time adaptive interventions based on passive data collection and hence with minimal participant burden.

## 1. Introduction

Overeating and unhealthy food choice have evolved into a major health system challenge, as prevalence of overweight and obesity continues to rise globally (1). Overeating is partially due to excessive availability of energy-dense, ready-to-eat, highly palatable foods. A key determinant of unhealthy food intake is *food craving*, the intense desire to consume a specific type of palatable food (2). Food craving covaries strongly with hunger around main mealtimes but can substantially deviate otherwise (3), like the craving for a palatable sweet desert after a filling savory main meal.

Being related to snacking (4), thoughts about eating (4), breaches of weight loss dieting (5, 6), and binge eating (7, 8), food craving is likely a pivotal construct in unhealthy and disordered (over)eating and thus a central target for intervention and prevention. Food craving can vary rapidly across the day and responds strongly to environmental temptations, such as to cues associated with food and eating (e.g., food outlets) or to (digital) food advertisements. Also, social context may matter as the presence of others can either facilitate or inhibit overeating (9). Yet, food craving can also covary with internal states such as emotions and stress (10, 11). Some situations such as noisy environments or episodes with frequent swift location changes might impact craving indirectly through its effects on stress or emotions (12), that, in turn, trigger cravings.

How could one intervene in a state that is as variable as food craving? Smartphone-delivered “just-in-time adaptive interventions” (JITAI) (13) intervene in real time and in everyday life, can adapt to the given (digital) context, and might thus be applicable to food cravings. Yet, to allow individuals to take preventive measures, i.e., to avoid certain cues or context, it would be useful to predict food cravings *ahead of time*, i.e., before craving translates into actual unhealthy overeating or even binge eating behavior. Thus, preventive JITAIs require prediction of food craving trajectories into the near future. Two types of studies can be delineated that attempt to do so: (a) ecological momentary assessment (EMA) studies and (b) smartphone data-based studies. Regarding the first case, EMA studies repeatedly assess not only the outcome variable (e.g., food craving) through smartphone-based self-report but also potential trigger states such as negative emotions, stress, various external events, and then predict the outcome variable from those trigger states. Spanakis et al. (14), for example, used 15 EMA questions assessed every 2 h (including food craving) for several weeks to develop decision trees that predict craving and choice of unhealthy foods. Large interindividual differences were noted by the authors and, thus, they used clustering algorithms to distinguish six types of eaters with different sets of predictor importance for unhealthy eating in each cluster. Forman et al. (15) and Goldstein et al. (16) used 21 EMA items to predict dietary lapses (eating more than planned) achieving an accuracy of 72%. Our own group used 18 EMA items in healthy individuals that were motivated to improve their diet (17). Using EMA items as predictors yielded an AUC of 0.63 in predicting food craving as rated on the next questionnaire, 2.5 h later. Interestingly, in our study, similar accuracies could be reached with only time- and smartphone-derived predictors, pointing to the potential of EMA-independent, purely passive predictor models.

The second type of prediction studies resort to sensor and smartphone usage data as “background data” that do not require active collaboration from the participants for predictor variable sampling. On the one hand, this has the clear advantage of strongly reducing the burden on participants. On the other hand, the clinical interpretability of the relationships between predictor variables and the dependent variable may be reduced as the predictor variables mostly serve as proxy variables for psychological or contextual states. Along these lines, Crochiere et al. (18) recently studied 23 adults with overweight/obesity who completed a 6-week commercial app-based weight loss program. They used 17 lapse-trigger EMA questions to predict subsequent dietary lapses (exceeding the intended eating goal in a weight watchers’ program) and achieved an accuracy of 70%. Using only four passive sensors (GPS, physical activity, sleep, and time), they reached an AUC of 61% and thus less than with EMA, but this was found to be clearly less burdensome by participants. Yet, their operationalization of dietary lapses does not directly speak to the experience of cravings (other reasons might lead to exceeding an eating goal), prediction accuracies were rather modest, and GPS tracking imposes privacy issues.

Thus, the present study set out to predict food craving ahead of time as a potential basis for preventive JITAIs using solely smartphone-derived, non-GPS predictor variables to allow a background assessment with minimal participant burden. Specifically, we used accelerometer, noise, and light sensors as well as phone usage patterns (notifications, screen activities) and time of day to predict upcoming food craving (assessed through EMA). In this work, we designed a strongly individualized machine learning approach, given the known interindividual differences in eating behavior, on the one hand, and of smartphone usage patterns, on the other. Specifically, we individualized the split point on which each individual's craving distribution was categorized as high or low and employed a data-driven selection of one out of six machine learning approaches. We then compared this against a prediction model based on past craving values but without sensor information. We expected to reach classification accuracies like ours [63%, Kaiser and Butter (17)] and others’ research (61%, Crochiere et al. (18)]. We further explored which prediction sensors were most useful and graphically illustrate representative cases.

## 2. Materials and methods

### 2.1. Participants

Participants included in the present secondary data analysis came from a larger randomized control trial (DRKS, DRKS00017493).^1^ Other parts of the data have previously been used in the studies by Pannicke et al. (19) and Kaiser and Butter (17). Participants were recruited to be motivated to maintain or reduce their body weight. Hence, the inclusion criterion was that participants agreed to one of the following two questions: (1) “Do you currently pay attention to your nutrition to maintain or reduce your body weight?” and (2) “Do you currently cut down on your food intake to maintain or reduce your body weight?”. Additionally, participants were only included if they owned an Android smartphone to run the application. From the control group of this intervention trial (no interventions delivered), we included 83 participants, out of which 27 participants were excluded from the analysis because of insufficient data (e.g., answering less than 50% of all EMA questionnaires, not providing processable sensor data) or technical problems, leaving the data of 56 participants for the analysis of the present study. Participants exhibited a mean age of 22.6 and were predominantly female (83%). All participants received oral and written information on the purpose of the study and signed an informed consent form approved by the Ethics Committee at the University of Salzburg, Austria.

### 2.2. Procedure

During individually arranged telephone calls, participants were instructed how to use the “SmartEater” application. The app was developed by the research group specifically for the purposes of conducting scientific studies by collecting passive smartphone background sensor data as well as EMA questionnaire data. The development was performed solely for the Android operating system as in this system the tracking of background data is much more flexible as compared to iOS devices. Three-day pre- and post-assessment EMA phases enclosed the main 14 day EMA phase (which served as the “treatment” phase for the active group with regular eating-related tips, respectively, as the control group) that served as the database for the current paper.

### 2.3. EMA measures

Participants received six signal-contingent EMA questionnaires per day in ±15 min time slots calculated from fixed time points (9:00 a.m., 11:30 a.m., 2:00 p.m., 4:30 p.m., 7:00 p.m., and 9:30 p.m.) for 14 consecutive days (i.e., 84 EMA questionnaires in total) by app notifications. All questionnaires were thus separated by semi-random time intervals of 150 (±15) min. Participants were able to answer questionnaires for up to 60 min after the initial notification of its availability. Additionally, participants were reminded about the availability of a new questionnaire every 15 min of this 60-min period. After this period, the questionnaire was marked as unanswered. Several psychological variables such as momentary affect, stress, and eating-related measures were assessed with at least 19 questions per prompt but were not used for the present study.^2^ However, one item queried momentary food craving (“How strong is your urge for specific, palatable food at the moment?”) on a horizontal visual analog scale from 0 (=not at all) to 100 (=very much) that served as a dependent variable.

### 2.4. Sensor data

In addition to the EMA functionality described above, the SmartEater application collected data of a multitude of sensors in the background out of which the following were used in this study: **Accelerometer:** every 5 min, the average change in acceleration was collected from raw accelerometer data where a principal component analysis was used to continuously (re-)identify the direction of strongest acceleration change; **Audio Volume:** mean, minimum, and maximum decibel (dB) values of 10 s recorded with the phone's microphone every 15 min; **Light:** mean, minimum, and maximum light values of 10 s recorded by the phone's light sensor every 15 min; **Notifications:** time and app name of incoming notifications of other smartphone applications. For privacy reasons, notification contents are neither read nor processed; **Screen-On-Time:** timestamps of when the screen was turned on or off; **Time of day:** while this input is not directly collected by a separate sensor, it is an additional entry of each sensor. The raw sensor data were first processed directly on the device before it is securely transmitted to the server backend in periodic time intervals for further storage and processing. The application does not require a constant internet connection for this functionality to work. Data are stored locally until a connection to the internet is re-established, and it is successfully sent to the server. There is no way known to us to test the sensor measurement precision of individual phones.

To compile the lagged explanatory variables from each usage or sensor data source for our models, we aggregated the data for time windows of 30 min. The windows’ boundaries are defined based on the time when a (craving) questionnaire was answered by a participant (T0). The first window starts at T0–150 min, the second window starts at T0–120 min, the third window at T0–90 min, and the fourth window at T0–60 min. The data starting at T0–30 min are not used for the prediction as we aim to be able to predict craving values 30 min into the future. This was the shortest time window that we considered sufficient for preparation of an intervention or a craving-preventive action in case of a real-time implementation of the present prediction approach.

### 2.5. Descriptive statistics

On average, the data set has about 70 (SD = 11.1, range 41–85) craving ratings per subject. The mean value of these craving values is 21.9 with a standard deviation of 26.7 (range: 0–100). It is worth mentioning that the craving data of most study participants exhibit a relatively severe skewness. The average skewness of the craving data is equal to 1.28 (SD = 0.98, range −0.97 to 3.26). Our train-test split of about 75% (10 days)/25% (4 days) results in test sets that average about 19.34 (SD = 3.07, range 12–25) data points.

The average craving ratings per person, as well as other statistical key figures, are presented in the Supplementary Material. Furthermore, on average, 84.8% (SD = 16.59, range 24.37%–99.76%) of all sensor data is available. The supplement also includes participant-specific breakdowns.

### 2.6. Individualized modeling

This study attempts to improve the prediction of food cravings through individualization. Individualization is performed with respect to three aspects: (a) individual separation of craving values in two classes (low and high) with three different thresholds, (b) individual decision whether to apply outlier detection and removal to the data, and (c) individual choice from six different prediction models. This procedure resulted in 36 (3 × 2 × 6) different possible combinations per study participant. From all these combinations, the best fitting individual combination of classification threshold, application of outlier detection, and choice of prediction model were determined as described in the following.

*Individual craving classes*. Classification algorithms require partitioning of numerical craving data into discrete classes. Due to strong individual skewness of the craving data (both positive and negative skew) craving ratings were classified as being either low or high using the 25%, the 50%, or the 75% quantile, generating three different splits for each participant. This approach gives the model selection procedure the chance to select individual thresholds that balance the classes in the data, thereby creating models with higher within-person generalization.

*Outlier treatment.* As self-reporting can be influenced by a multitude of factors, possible outliers, which do not fit into the general craving patterns of a person, have to be considered. Therefore, we allowed predictions with and without outlier detection and removal based on the isolation forest (20) method.

*Individual algorithm selection and training/test data split.* Training and test data were split in an approximate 75% (10 days)/25% (4 days) ratio. The training/test data split was done day-wise (not signal-wise) to retain typical diurnal patterns. Furthermore, selecting the last four consecutive days or selecting random days resulted in a more imbalanced test set for most participants. Supplementary Appendix D illustrates this issue. Based on these observations, we refrained from using a cross-validation approach and resorted to a single train-test split. The selection of the days defining the test set was performed by a “greedy algorithm.” This algorithm iteratively selects 4 days for the test set. In each step, that single day is chosen that minimizes the difference between the growing test set and the complete set with respect to the high vs. low craving ratio.^3^ The corresponding python code is provided in Supplementary Appendix E.

All 36 models were then applied to the remaining 10 days (individualized training data set). We used Logistic Regression, Decision trees, Support Vector machines, ADA-Boost, XG-Boost, and a Multi-layer Perceptron and thus some of the most popular algorithms applied to such use cases [Zhang et al. (21)]. After the training phase, all of these 36 models were applied to the individualized test set. This led to 36 results per participant. The model that achieved the highest score on the test set, i.e., the one that generalized best from all 36 models, was selected. All further analyses were then conducted with this model for the given participant.

### 2.7. Comparison against a baseline model

To measure the craving-related information that sensors and usage patterns are able to add, the individualized prediction approach was further validated against a “baseline model” without sensor data. For that baseline model, we chose a time series prediction based on the past four consecutive craving values (which might include values of the previous day). We applied the same preprocessing and prediction pipeline as above, except that no individual craving split point was applied. Thus, we again trained six algorithms with or without outlier detection yielding 12 AUC values, out of which the highest was chosen. The confidence intervals for the individual AUC scores were calculated according to the method of Hanley and McNeil (22) and are provided in the Supplementary Material.

### 2.8. Feature importance and illustrative cases

To obtain an overview of the general importance of each feature for prediction, permutation importance (23) was used on each participant and then averaged for descriptive purposes. Feature importance is defined through the loss of AUC if feature values are randomly shuffled over the columns, thereby breaking any (if existent) relationship between the feature and the dependent variable. This concept is called permutation importance and was first introduced by Breiman in 2001 (23). Due to the inherently random nature of this measure, the feature importance was calculated 50 times and then averaged. Furthermore, SHapley Additive explanation (SHAP) (24) plots were used to illustrate the contribution of different features to classifications for three exemplary cases. In these plots, predictors are ranked from the strongest predictor (top) to the weakest predictor (bottom). The horizontal deviation shows how each predictor contributes to the separation of high vs. low craving scores. Each row of data in the test data set containing the respective values of the features corresponds to a line in the plot. For each data point, the corresponding SHAP value is calculated, and the sign and the magnitude of this value determine the strength of the deflection of the line. Depending on the underlying prediction model, it is possible that there are identical lines. The intersection of each of these lines with the x-axis at the top of the graph, determined by the sum of all SHAP values, corresponds to the predicted probability indicating either a high or low craving value. The magnitude of the slope for each feature visualizes the importance of that feature for the prediction. The order in which the features are listed on the y-axis corresponds to the sum of the magnitudes of their SHAP values over all data points. This means that the features are ordered according to the strength of their influence/importance.

## 3. Results

### 3.1. General evaluation and comparison to baseline model

AUC (22) and Brier (25) scores were used for evaluation. AUC scores averaged at 0.78 (SD = 0.10, range 0.58–0.97), while Brier scores averaged at 0.23 (SD = 0.05, range 0.11–0.41). In comparison, the baseline model achieved an average AUC score of 0.64 (SD = 0.11, range 0.39–0.92), i.e., 0.14 less. The average 90% confidence interval for the sensor-based model is 0.59–0.96 and that of the baseline model is 0.41–0.88. We also performed a Mann–Whitney *U*-test between the results of the two models that were highly significant, *U* = 2587.5, n1 = n2 = 56, *p *= 4.3 × 10^−10^ two-tailed, median of the sensor model = 0.77, and median of the baseline model = 0.64.

**Figure 1:**
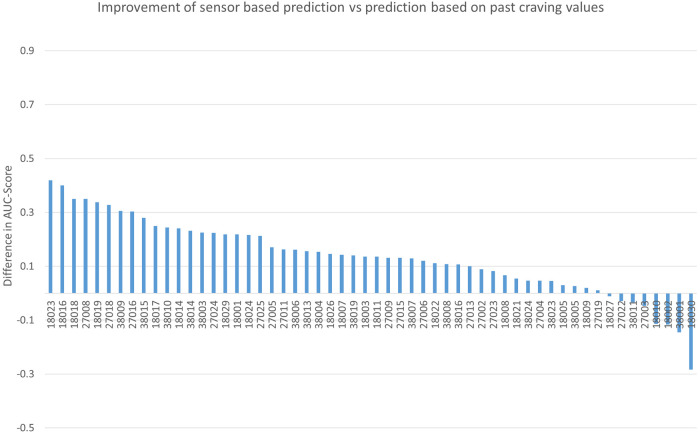
The difference in individual AUC scores between the sensor-based prediction (without previous craving values) vs. the prediction solely based on past craving values. AUC, area under the curve.

The results of the baseline model suggest that craving time course can be predicted to some degree from previous values and we are thus confident that the baseline model is a fair alternative against which the sensor model has to compete. Figure 1 depicts the advantage (or disadvantage) of the sensor model against the baseline model across all participants. It is evident that for the majority of participants (48 of 56, 85%), sensor data prediction outperformed the baseline prediction.

### 3.2. Feature importance

To obtain an overview of the general importance of each feature for prediction, permutation importance (23) was used on each participant and then averaged for descriptive purposes.

**Figure 2:**
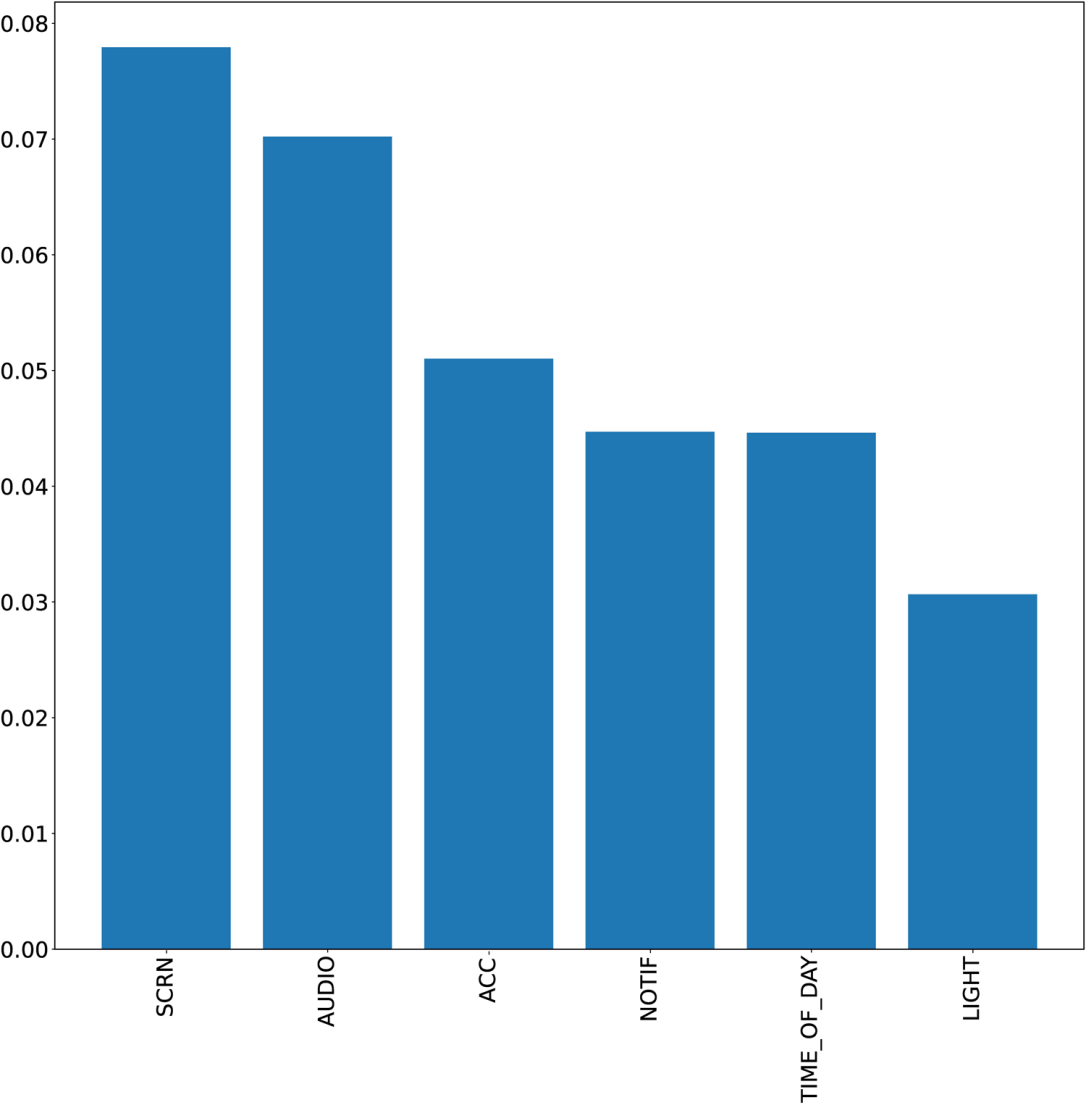
Permutation importance of the utilized features. The y-axis corresponds to the loss in AUC if this feature is permuted. AUC, area under the curve.

Figure 2 depicts the importance of the utilized features. The three most important features were SCREEN, AUDIO, and ACC. The decrease in importance over all features is relatively constant; thus, each feature seems to have positive contributions to the prediction at least in a subset of participants.

### 3.3. Feature importance assessed with SHAP scores

**Figure 3:**
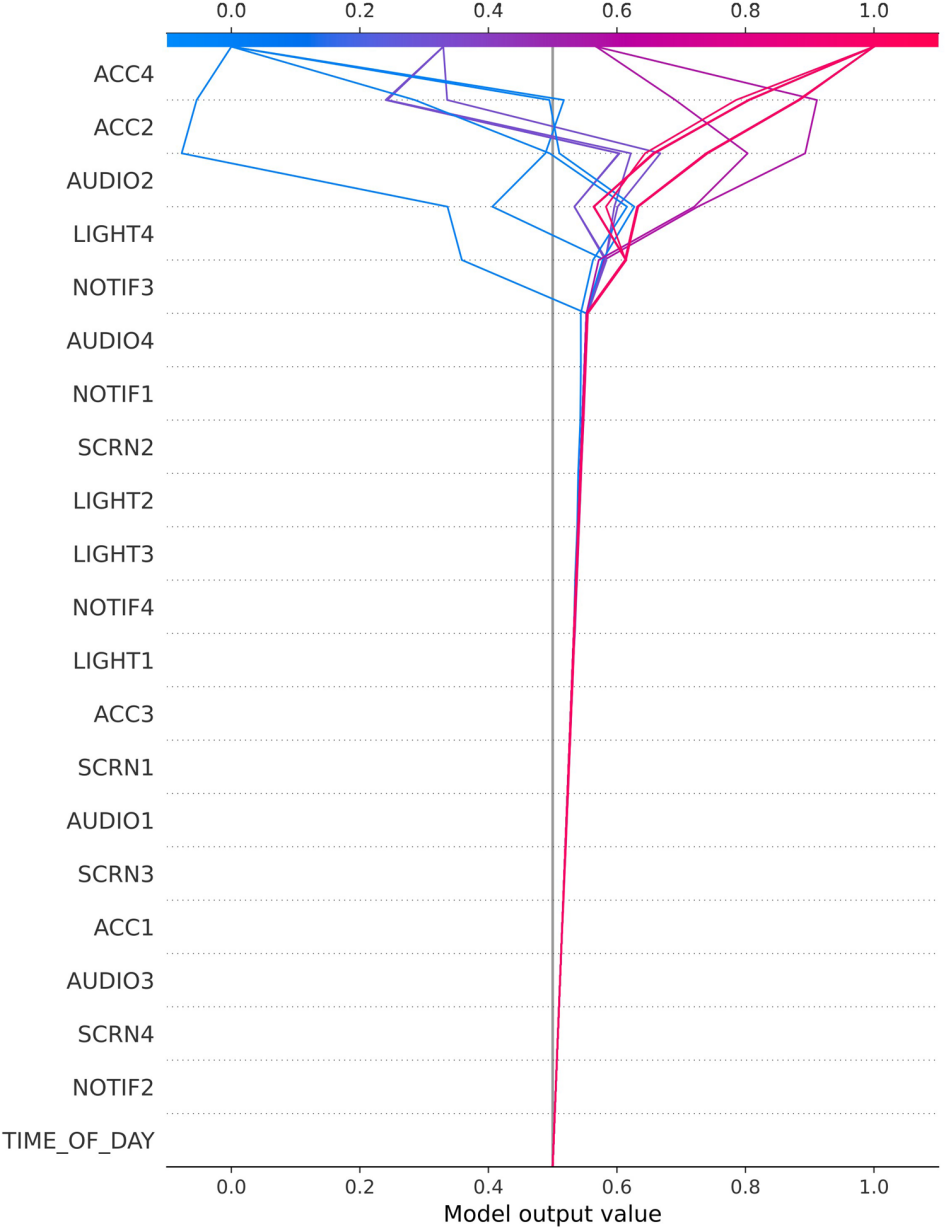
SHAP plot for user 27006.

**Figure 4:**
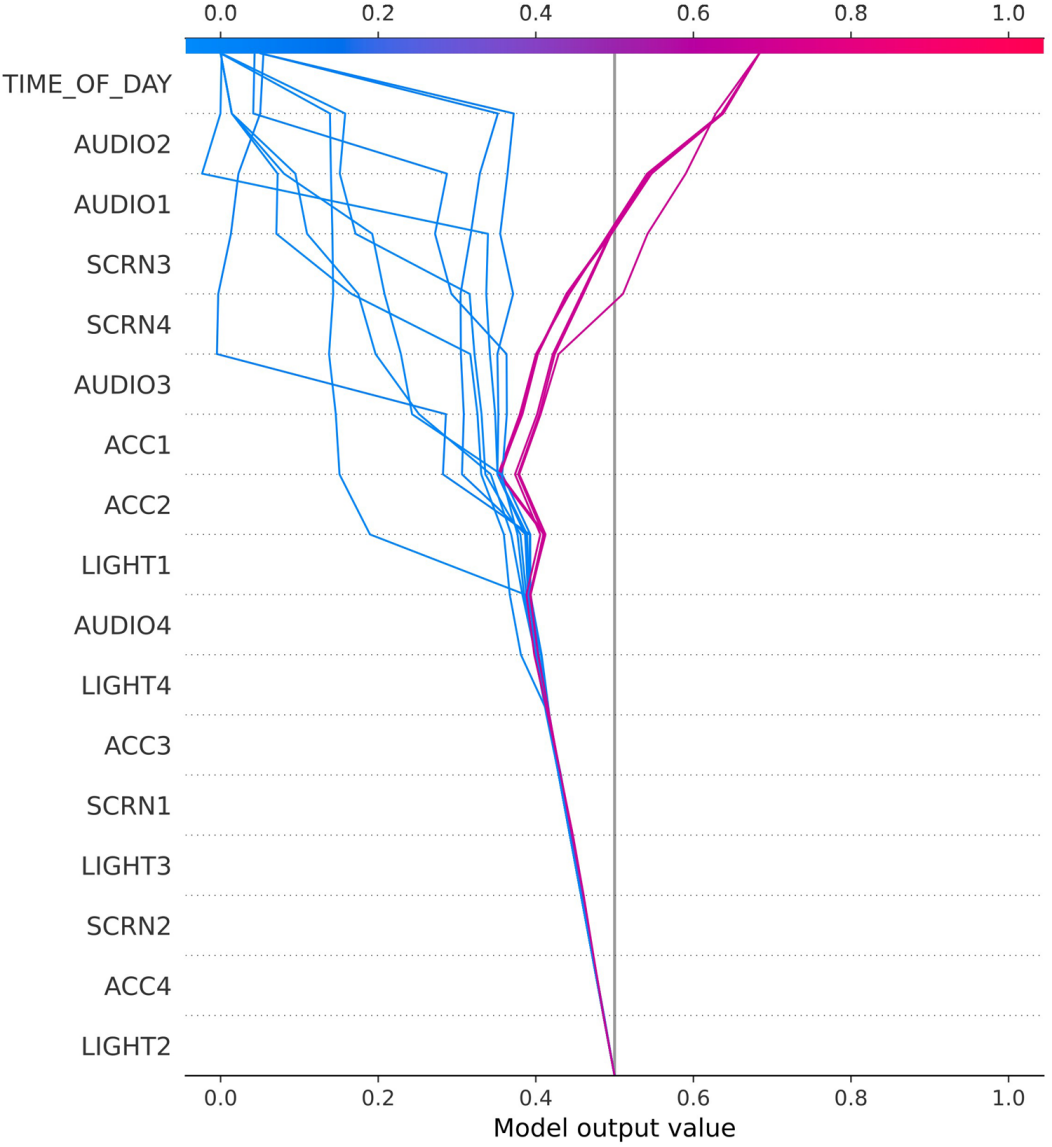
SHAP plot for user 38016.

**Figure 5:**
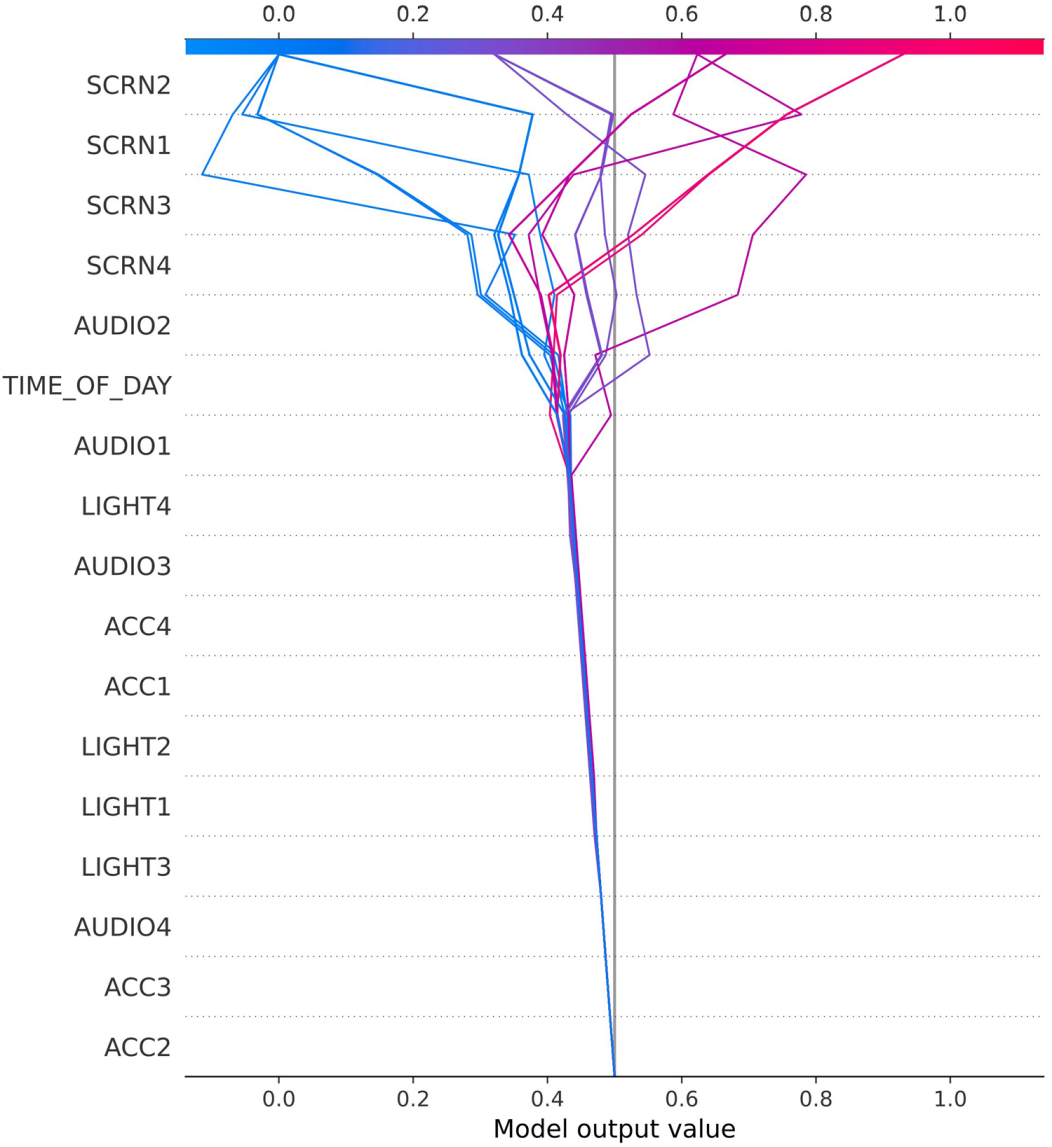
SHAP plot for user 38015.

As outlined in the introduction, smartphone usage, digital environments, and craving experience differ strongly between individuals and therefore call for an individualized analytical approach. To depict some of these between-participant differences and to illustrate the decision-making process of the prediction models, Figures 3–5 display three exemplary SHAP (24) plots.

The SHAP decision plot for study participant 27006 in Figure 3 illustrates the importance of movement (ACC) for separating high from low craving scores, followed by Audio and Light. These might be proxies for external situational context changes that triggered craving episodes in this user, e.g., when returning home from work (putting smartphone on the table) and experiencing craving due to availability of tempting foods at home. By contrast, craving values of user 38016 (Figure 4) seemed to be sensitive to time of the day, with some daytimes being highly predictive of—relatively few—high craving scores. The audio predictors might indicate environmental context changes (e.g., outside–inside transitions) and screen activities point to smartphone use.

User 38015 (Figure 5) presents yet another type of feature that is related to craving. Here screen activity is most prominent, which could be translated to the simplified formula: smartphone usage = craving.

## 4. Discussion

The aim of this study was to explore whether food cravings could be predicted through passively collected smartphone sensor and usage data only using individualized prediction approaches. The information conveyed in the smartphone data increased craving prediction accuracy by 14% over accuracies derived from past craving data. Advantages for sensor-based models over craving-based models were seen in 85% of participants.

Based on our results, a maximal accuracy of 78% seems possible even though this might contain some residual degree of overfitting. We accepted some degree of uncertainty here as our 14 days of data did not allow a split into independent training, validation, and test datasets that would each have reasonable distributions of low vs. high craving data (see Supplementary Appendix). For reference, predictions have reached higher values with other problematic health behaviors, e.g., an accuracy of 87% was reached in the prediction of smoking using only accelerometer and GPS data (26). An accuracy of 90.9% was reached in the detection of alcohol consumption (27) using sensor data [day and time, accelerometer, gyroscope, communication behavior, and data indicating psychomotoric constraints (e.g., typing speed)]. The fact that food craving is a fleeting subjective state and not an observable behavior may explain the typically lower prediction accuracy for food cravings compared to other health behaviors.

Our approach always requires individual model training on at least 14 days of data before actual predictions of future craving values can be made. For faster predictions based on passive sensing—i.e., without a full 2 weeks of craving recording, one would have to classify participants into groups with associated pretrained prediction models based on single questionnaires or at least fewer days of data recording. This relates to the well-known cold-start problem in recommended systems. A classification in the groups was described in detail by Spanakis et al. in 2017 (14). Our SHAP decision plots illustrated these interindividual differences, possibly stemming from both differences in smartphone use and food craving experience. Despite the differences in feature importance for our three exemplary users, between-participant clustering might be possible to allow for use of the same model for participant groups. This assumption is based also on the combinatorial argument that by using six types of sensor data there are only 15 different combinations to choose the two most important types of data per participant.

Regarding predictors, screen-on-time was a highly relevant predictor contributing positively to food craving prediction in many individuals. This might be related to the associated active interaction of the user with the smartphone as well as its high frequency. Environmental noise and movement proved useful across many users. This is also supported by data from earlier studies where especially noise related to the perceived stress scale (12). Usage of GPS data may prove useful in future studies, albeit privacy issues with these data are very sensitive and must be solved by local on-device classification of the location data, thereby eliminating the need for coordinate storage.

The present results have clear practical and clinical implications: being able to derive potential real-time predictions of upcoming food craving peaks can be used to trigger interventions on such states. Since food craving is related to snacking (4), breaches of weight loss dieting (5, 6) and binge eating (7, 8) such as in the moment interventions could have a significant impact. Interventions could include mental distraction (food-unrelated imagery), substitution of unhealthy by healthy snacks, goal reminders, and “urge surfing” techniques, among others (28, 29). Such interventions could be applied in healthy individuals with an intention to increase healthy eating as well as to patients with eating and weight disorders in outpatient care (where binge eating might be predicted) (30). Short motivational SMS-based interventions have been successfully applied already a decade ago (31). Additionally, prediction-timed interventions are available (15). Yet, final evaluations of such protocols in micro-randomized and classical randomized controlled trials are still pending.

## 5. Limitations

Conclusions are limited by the fact that we had insufficient data to use a validation and a test set as compared to using only one test set, which might result in a residual amount of overfitting. Furthermore, limitations are inherent in the sample, so generalization to other populations carries some uncertainty. Finally, craving, though a relevant mediator of unhealthy eating, is not an actual behavior. Future research would need to demonstrate whether JITAIs should best be targeted at craving or actual eating behavior.

## Data Availability

Raw data is not available in compliance with Austrian privacy laws. Further inquiries should be sent to the corresponding author/s.

## Footnotes

^1^Primary outcomes of the trial were intervention-based changes in EMA eating behavior, especially regarding successful implementation of one's intentions to eat “in line with one's goal.”

^2^Primary analyses mainly used the eating-related variables to examine changes across the intervention period in comparison to the control group.

^3^To ensure that our “greedy” procedure does not result in an unrealistic evaluation, we recalculated the results using the last 4 days as a test set. The mean AUC of these models is 0.78. However, a closer inspection showed that in this case many test sets were highly imbalanced (four participants even had only one craving class in the test set). This severe imbalance results in models that predict only one class and are, therefore, of no practical use.
