# Supplementary material for: Predicting food craving in everyday life through smartphone-derived sensor and usage data
Source: Front Digit Health. 2023 Jun 26;5:1163386. doi: 10.3389/fdgth.2023.1163386 (PMC10331138; doi:10.3389/fdgth.2023.1163386)
Supplement: Supplementary file 5 [file Datasheet5.docx]

# Appendix A


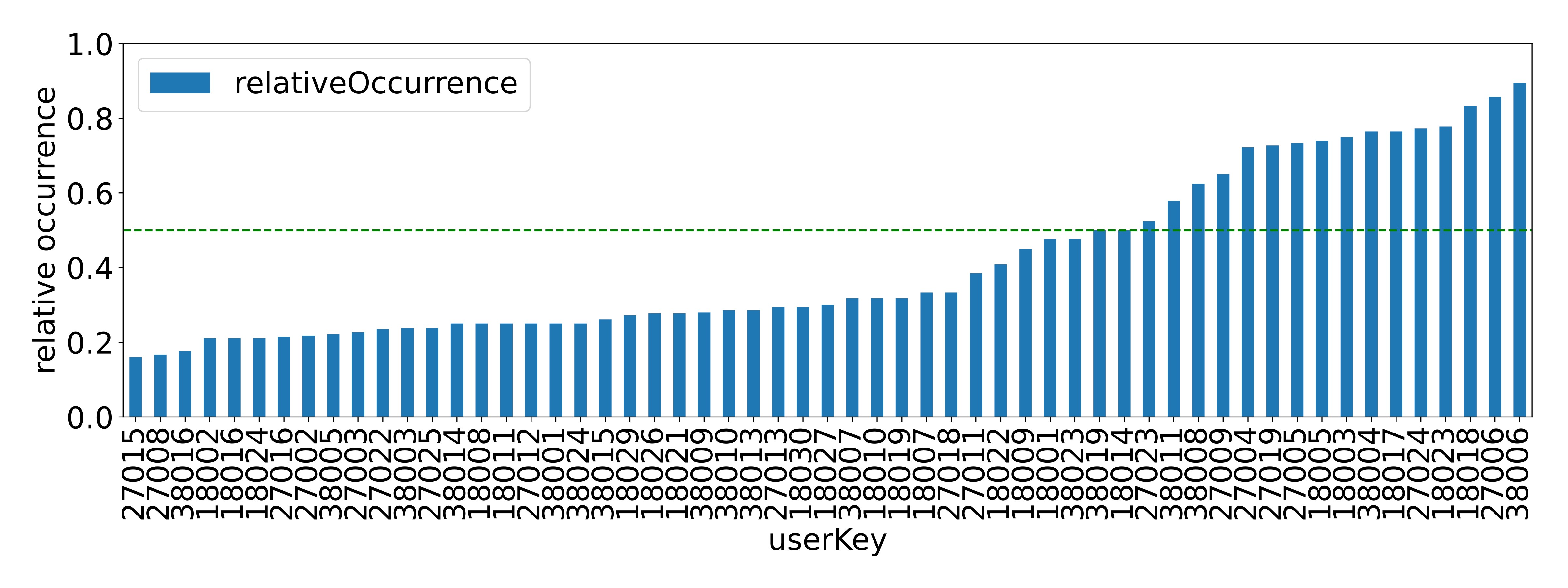


Figure 6: This figure illustrates the relative frequency of the label HIGH in the test data set.

To ensure that the score achieved here did not occur due to a severe predominance of one class in the test set, which could theoretically occur due to the individualization process, figure 6 visualizes the relative frequency of the label HIGH in the test set. Figure 6 shows that for 38 out of 56 participants the relative frequency of the class HIGH is less than 50%. Thus, there is a certain bias towards the individualized models, which use the third quantile as the splitting point. However, this bias can be partly attributed to the skewness of the underlying data. The average skewness of the underlying craving data is 1.28 (SD = 0.98, range -0.79 - 3.26). Thus, the data is relatively skewed, both positively and negatively, which then also leads to an imbalanced distribution.

# Appendix B

Tables 1 and 2 present a breakdown of the results with respect to the individualized models. Table 1 shows how often an outlier removal technique was used in the best model, as well as the average AUC score. Table 2 breaks down the results to the prediction model used.

|  | used in # models | avg. AUC | SD | Min | Max |
| --- | --- | --- | --- | --- | --- |
| Isolation Forest | 21 | 0.78 | 0.09 | 0.58 | 0.96 |
| None | 35 | 0.77 | 0.1 | 0.58 | 0.97 |

Table 1: This table presents a breakdown of the results obtained according to the outlier removal technique used.

|  | used in # models | Avg. AUC | SD | Min | Max |
| --- | --- | --- | --- | --- | --- |
| Dec. tree | 3 | 0.79 | 0.04 | 0.72 | 0.82 |
| Logistic Reg. | 8 | 0.76 | 0.1 | 0.63 | 0.95 |
| XG-Boost | 8 | 0.74 | 0.09 | 0.58 | 0.89 |
| SVM | 10 | 0.81 | 0.09 | 0.67 | 0.97 |
| ADA-Boost | 11 | 0.79 | 0.08 | 0.69 | 0.92 |
| MLP | 16 | 0.77 | 0.11 | 0.58 | 0.96 |

Table 2: This table presents a breakdown of the results obtained according to the prediction model used.

# Appendix C

Table 3 provides an overview of the number of study participants for whom a specific feature is the most important for prediction. The term "most important" here implies that the feature was at least one standard deviation above the mean of the permutation importance.  In the case that two or more features were further than one standard deviation away from the mean, only the feature with the largest distance was considered important. The case where more than one feature was one standard deviation above the mean occurred in six study participants, for one study participant no single individual feature was important. Additionally, this table also presents how often a feature had no added value for the prediction, i.e. a permutation importance of zero. The importance of individual features for specific individual users varies, as indicated by table 3. This can probably be explained by the varying uses of the smartphone. For example, placing the smartphone on the table and using it mostly in this position means that the accelerometer does not provide much information about different life contexts which may or may not lead to craving. The features SCRN and NOTIF, for example, are not affected by this. This behavior is also reflected in the user specific SHAP plots shown in section 4.3. Some models make their predictions based on relatively few features; others use almost all available features.

|  | Was most important feature # times | Was zero # times |
| --- | --- | --- |
| ACC | 8 | 6 |
| SCRN | 12 | 4 |
| AUDIO | 15 | 5 |
| LIGHT | 5 | 8 |
| NOTIF | 6 | 12 |
| TIME OF DAY | 9 | 9 |

Table 3: This table presents an overview about how often which feature was the most important and how often it had zero importance.

# Appendix D

Figures 7 and 8 illustrate the difference in relative occurrence of the class label “HIGH” between the whole dataset and the test set (last 4 days as test set in figure 7 and the test set chosen by the greedy algorithm in figure 8). Note that in the case of the test set consisting of the last 4 days, four participants had to be removed because the class "HIGH" did not appear once.


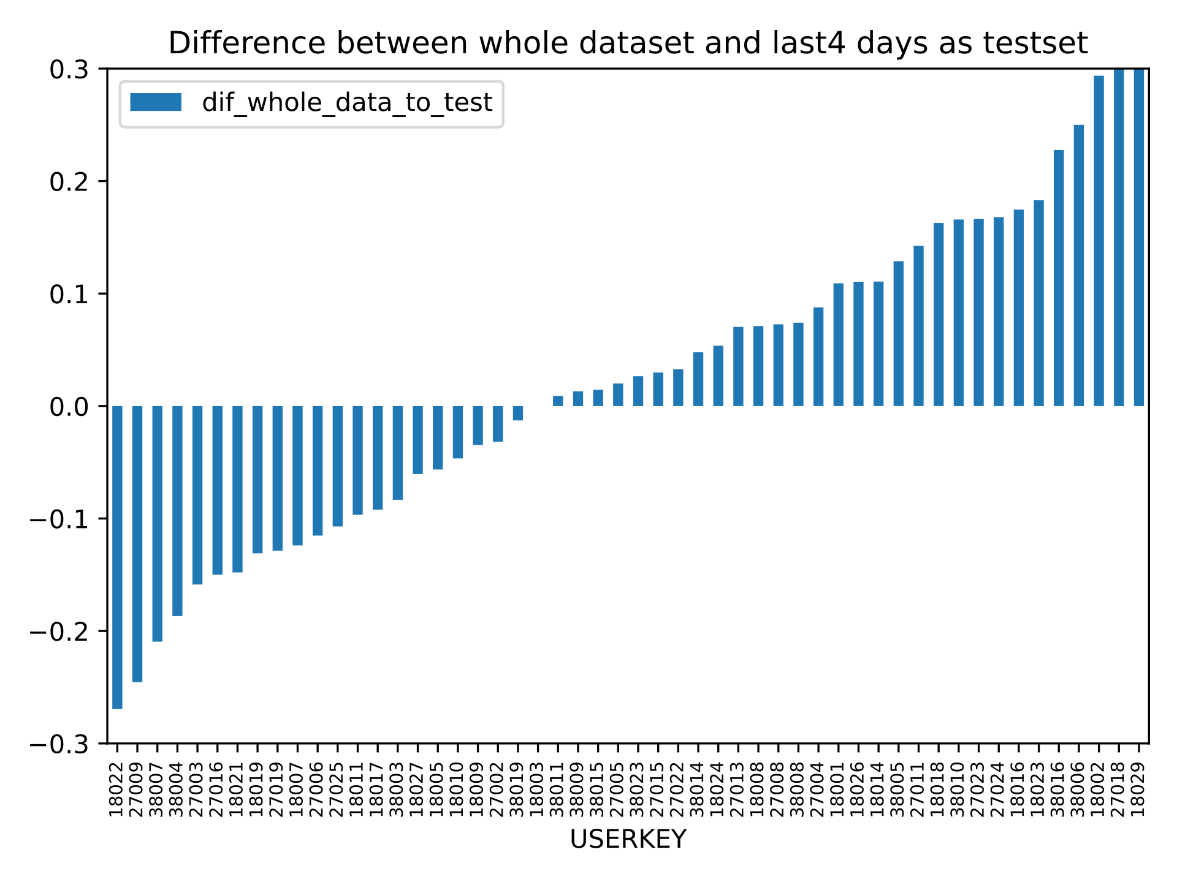


Figure 7: Depicting the difference of the relative occurrence of the class "HIGH” between the whole dataset and the test set consisting of the last four days


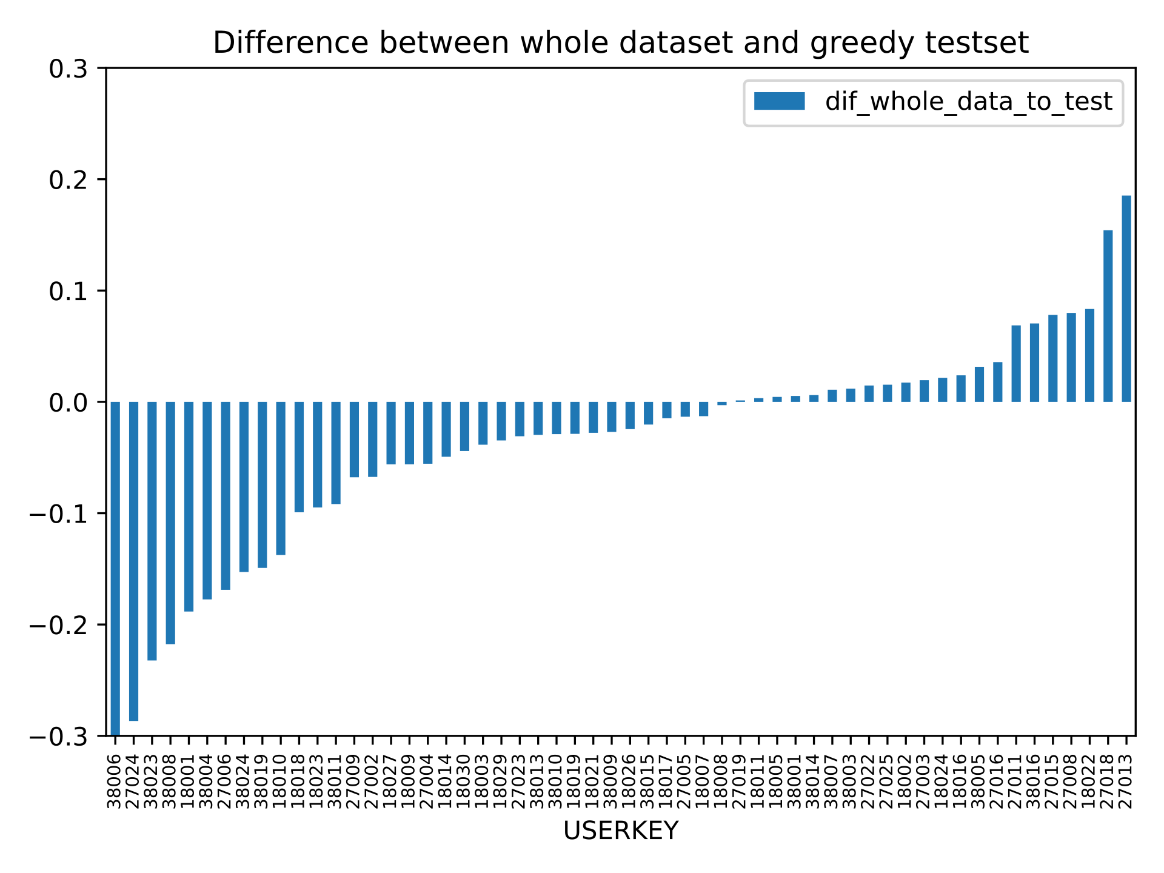


Figure 8: Depicting the difference of the relative occurrence of the class "HIGH” between the whole dataset and the test set chosen by the greedy algorithm

# Appendix E:

Python code for the greedy algorithm:

meanRelFrequency = result["rel frequency"].mean()
test = DataFrame(columns=result.columns)
# 4 days for test data --> leaves 10 days train data
# greedy algorithm
# always choose next day so relative frequency of HIGH values gets somewhat the same in test-set

for i in range(0, days):
 curSum = test["rel frequency"].sum()
 curLen = len(test)
 result["futureMeanFreq"] = ((curSum + result["rel frequency"]) / (curLen + 1))
 result["SortKey"] = np.abs((meanRelFrequency - result["futureMeanFreq"]))
 result = result.sort_values("SortKey")

 result.drop(axis=1, labels="SortKey", inplace=True)
 result.drop(axis=1, labels="futureMeanFreq", inplace=True)
 next = result.iloc[:1]
 result.drop(next.index, inplace=True)
 test = pd.concat([test, next], ignore_index=True)

[Figure Captions]

Figure1: This figure depicts the difference in individual AUC-Scores between the sensor-based prediction (without previous craving values) vs. the prediction solely based on past craving values.

Figure2: Permutation importance of the utilized features. The y-axis corresponds to the loss in AUC if this feature is permuted.

Figure 3: Shap plot for user 27006

Figure4: Shap plot for user 38016

Figure5: Shap plot for user 38015

Figure6: This figure illustrates the relative frequency of the label HIGH in the test data set.

Figure7: Depicting the difference of the relative occurrence of the class "HIGH” between the whole dataset and the test set consisting of the last four days

Figure 8: Depicting the difference of the relative occurrence of the class "HIGH” between the whole dataset and the test set chosen by the greedy algorithm

[Tables]

Table 1: This table presents a breakdown of the results obtained according to the outlier removal technique used.

|  | used in # models | avg. AUC | SD | Min | Max |
| --- | --- | --- | --- | --- | --- |
| Isolation Forest | 21 | 0.78 | 0.09 | 0.58 | 0.96 |
| None | 35 | 0.77 | 0.1 | 0.58 | 0.97 |

Table 2: This table presents a breakdown of the results obtained according to the prediction model used.

|  | used in # models | Avg. AUC | SD | Min | Max |
| --- | --- | --- | --- | --- | --- |
| Dec. tree | 3 | 0.79 | 0.04 | 0.72 | 0.82 |
| Logistic Reg. | 8 | 0.76 | 0.1 | 0.63 | 0.95 |
| XG-Bboost | 8 | 0.74 | 0.09 | 0.58 | 0.89 |
| SVM | 10 | 0.81 | 0.09 | 0.67 | 0.97 |
| ADA-Boost | 11 | 0.79 | 0.08 | 0.69 | 0.92 |
| MLP | 16 | 0.77 | 0.11 | 0.58 | 0.96 |

Table 3: This table presents an overview about how often which feature was the most important and how often it had zero importance

|  | Was most important feature # times | Was zero # times |
| --- | --- | --- |
| ACC | 8 | 6 |
| SCRN | 12 | 4 |
| AUDIO | 15 | 5 |
| LIGHT | 5 | 8 |
| NOTIF | 6 | 12 |
| TIME OF DAY | 9 | 9 |
